# Correlation Between Metabolic Syndrome, Periodontitis and Reactive Oxygen Species Production. A Pilot Study

**DOI:** 10.2174/1874210601711010621

**Published:** 2017-12-12

**Authors:** Romeo Patini, Patrizia Gallenzi, Gianrico Spagnuolo, Massimo Cordaro, Monica Cantiani, Adriana Amalfitano, Alessandro Arcovito, Cinzia Callà, Gertrude Mingrone, Giuseppina Nocca

**Affiliations:** 1 Institute of Dentistry, Università Cattolica del Sacro Cuore, Largo Francesco Vito - 00168 Rome , Italy; 2 Department of Neurosciences, Reproductive and Odontostomatological Sciences, Università di Napoli Federico II, *via* S. Pansini, 5-80131 Naples, Italy; 3 Institute of Biochemistry and Clinical Biochemistry, Università Cattolica del Sacro Cuore, Largo Francesco Vito - 00168 Rome , Italy; 4 Department of Internal Medicine, Università Cattolica del Sacro Cuore, Largo Francesco Vito - 00168 Rome , Italy; 5 Istituto di Chimica del Riconoscimento Molecolare, CNR, Largo Francesco Vito - 00168 Rome , Italy

**Keywords:** Metabolic syndrome, Periodontitis, Obesity, ROS metabolism, Chemiluminescence, Leukocytes

## Abstract

**Background and Objective:**

Metabolic syndrome (MetS) is associated with an increased risk of periodontitis even if the mechanism is unknown. Since both MetS and periodontitis are characterized by an alteration of inflammation status, the aim of this pilot study was to determine if differences in ROS metabolism of phagocytes isolated from (A) patients with MetS, (B) patients with both MetS and mild periodontitis, (C) healthy subjects and (D) normal weight subjects with mild periodontitis, were present.

**Methods:**

ROS metabolism was studied by a Chemiluminescence (CL) technique: the system was made up of luminol (100 nmol/L) and cells (1 × 10^5^) in the presence or absence of stimulus constituted by opsonized zymosan (0.5 mg). The final volume (1.0 mL) was obtained using modified KRP buffer. ROS production was measured at 25°C for 2 h, using an LB 953 luminometer (Berthold, EG & G Co, Germany). All the experiments were performed in triplicate.

**Statistical Analysis:**

All results are mean ± standard deviation (SD). The group of means was compared by the analysis of variance "(ANOVA)". A value of *p* < 0.05 was considered significant.

**Results:**

Results showed that basal ROS production (both from PMNs and from PBMs) of groups A, B and D was increased with respect to that obtained from group C (*p* <0.05).

**Conclusion:**

These results are congruent with literature data, although the actual clinical relevance of the phenomenon remains to be evaluated.

## 1 INTRODUCTION

Metabolic Syndrome (MetS) comprises a set of risk factors for cardiovascular disease and type 2 diabetes mellitus [1, 2]; these risk factors are obesity, impaired glucose tolerance, hyperinsulinemia, dyslipidemia, pro-inflammatory state, *etc*., [3]. In addition, recent clinical studies have provided evidence that MetS increases with age [4] and it, as well as obesity is associated with an increased risk of periodontitis [5-9]. Since both obesity and MetS are characterized by lipid metabolism disorders, it is possible to hypothesize that this alteration increases the risk of developing periodontal disease [10], that is one of the most common chronic infection in the world [11]. In a recent paper [10], Li *et al.* showed that MetS is associated with increased periodontal inflammation induced by lipopolysaccharide (LPS) in Zucker fat rats (a model of MetS) with respect to control lean rats. In the same paper, the role of palmitic acid and LPS in periodontitis is also reported. More generally, it is possible to assume that the association between obesity and periodontal diseases is probably based on the effect of pro-inflammatory cytokines (*i.e.*Tumor necrosis factor-α-TNF-α-;granulocyte/ macrophage colony-stimulating factor-GM-CSF*-,etc.*) released by adipose tissue.

LPS – as well as pro-inflammatory cytokines, phorbol-12-myristate-13-acetate (PMA), and N-formylmethionyl leucyl phenylalanine (fMLP) is involved in Reactive Oxygen Species (ROS) production, through the activation of p47phox (a subunit of NADPH oxidases) by phosphorylation catalyzed by Protein Kinase C (PKC). Membrane NADPH oxidases are present in a variety of cells like granulocyte polymorphonucleates (PMNs), peripheral blood monocytes/macrophages (PBMs) system and endothelial cells [12].

The classical NADPH oxidase present in phagocytes consists of two membrane subunits (gp91phox and 22phox, forming flavocytochrome b558 complex), the active form of the enzyme is obtained when the cytosolic subunits p47phox, p40phox, p67phox, and Rac1 are activated and translocated to plasma membrane [13]. Only in the active form, the enzyme is able to transfer electrons from NADPH to O_2_ forming O_2_**^-.^** which is transformed into H_2_O_2_. PMNs are able to transform H_2_O_2_ in HOCl by the enzyme myeloperoxidase [14].

To explain the relationship between MetS, inflammation and periodontitis, the aim of this pilot study was to determine if differences in ROS metabolism of phagocytes isolated from (A) obese patients with MetS (B) obese patients with both MetS and mild periodontitis, (C) healthy subjects and (D) normal weight subjects with mild periodontitis, were present.

This type of study, which analyzes the systemic basis of oral cavity pathologies, is becoming more common as scientists are interested in the dental field [15] and in the multidisciplinary approach required in the treatment of patients with special needs [16]. This is because of the spread of new and sensitive techniques for the diagnosis of oral cavity pathologies [17].

## 2 MATERIALS AND METHODS

### 2.1 Patients

The sample of this pilot study was made up of four different groups (A, B, C, D):

Group A involved 2 MetS obese patients without any form of periodontal disease.

Group B involved 2 MetS obese patients with a mild form of periodontal disease.

Group C included 4 normal weight subjects without any form of periodontal disease.

Group D included 3 normal weight patients with a mild form of periodontal disease.

Patients belonging to groups A and B were consecutive patients admitted to the department of internal medicine of “Fondazione Policlinico Universitario Agostino Gemelli” teaching hospital to keep their obesity status under control with regular blood tests and specialist visits. They were selected according to the following inclusion criteria: over 40 years of age, Body Mass Index (BMI) over 40, absence of diabetes or any cardiac disease, and non-smokers or ex-smokers for more than 10 years. Patients who formed groups C and D were consecutive patients attending the dentistry institute of “Fondazione Policlinico Universitario Agostino Gemelli” teaching hospital for dental check-ups and treatments. Among these, 7 patients with age between 40 and 55, with the absence of diabetes or any cardiac disease, BMI lower than 30 and non-smoker or ex-smokers for more than 10 years were selected. In order to avoid confounding effects of ulcerative-erosive lesions of oral mucosa [18], benign [19, 20] and malignant [21] neoplasms, cystic lesions [22] and immunological diseases [23] on immunoinflammatory and hormonal response patients were selected in the absence of such conditions. For this purpose, patients underwent a clinical and radiographic dental check-up before inclusion in the present study. None of the study participants had received in the previous two months any steroidal or immunosuppressive drug which could influence immune and hormonal response. The study was conducted according to the 1975 Helsinki Declaration, as revised in 2000 and underwent local Ethics Committee evaluation (“Fondazione Policlinico Gemelli” Prot. N°0027002/17); written informed consent was obtained from every patient. Blood samples were collected in fertile women during the follicular phase. All patients (4 female and 7 male) underwent a periodontal examination in which Periodontal Screening and Recording (PSR) was performed. Patients were divided according to the periodontal status following the results of the PSR: codes 0-1-2 were considered as a state of general periodontal health whereas code 3 was associated to a mild form of periodontal disease. Only patients with homogenous PSR codes in all sextants were included. Based on BMI, patients were divided into obese (BMI > 40) or normal weight (BMI < 30). Demographic characteristics of patients included in this study are presented in Table **1**.

### 2.2 Polymorphonucleocytes (PMNs) Isolation

Venous blood (10 mL), obtained from each volunteers, was diluted with physiological solution (10 mL). Dextran in physiological solution (6%, 4 mL) was then added to enhance the sedimentation rate of erythrocytes at 1 × *g*. After 30 min, the white blood cells suspension was centrifuged on Lymphoprep (Pharmacia, Sweden), according to the manufacturer’s instructions, to remove the contaminating mononuclear cells, and finally the pellet underwent hypotonic lysis to remove erythrocytes. The recovered PMNs were washed three times and resuspended in Krebs Ringer phosphate (KRP) solution (200.000/mL, pH 7, 4).

### 2.3 Peripheral Blood Monocytes (PBMs) Isolation

Lympho-monocytes were isolated through lymphoprep (Pharmacia, Sweden) density gradient centrifugation.

Lympho-monocytes, suspended in DMEM (Dulbecco’s modified Eagle medium) with HEPES (10 mmol/L), glucose (1.0 g/L), NaHCO_3_ (3.7 g/L), penicillin (100 Units/mL), streptomycin (100 μg/mL) and 10% Fetal Calf Serum (1 × 10^6^ cells/mL), were put into luminometer vials to allow the adhesion (37°C, 5% CO_2_ humidified atmosphere, 1 h). Non-adhered cells were then gently removed by three washes with modified Krebbs–Ringer phosphate (KRP) buffer and counted by Nucleocounter (Sartorius Stedim S.P.A, Firenze, Italy) to calculate, by subtraction from the whole, the number of adhered cells [14]: the latter were mainly monocytes (> 90%), as determined using the aspecific esterase test, a typical monocyte enzyme [24].

### 2.4 ROS Metabolism of Leukocytes

ROS metabolism was studied by a Chemiluminescence (CL) technique: the system was made up of luminol (5-amino-2,3-dihydro-1,4- phthalazindione, 100 nmol/L) and cells (1 × 10^5^) in the presence or absence of stimulus constituted by or 150 nmol/L of phorbol 12-myristate 13-acetate (PMA) [24]. The final volume (1.0 mL) was obtained using modified KRP buffer. ROS production was measured at 25°C for 2 h, using an LB 953 luminometer (Berthold, EG&G Co, Germany). All the experiments were performed in triplicate.

### 2.5 Statistical Analysis

All the results are expressed as mean ± standard deviation (SD). The group of means was compared by the analysis of variance "(ANOVA)", followed when appropriate by a multiple comparison of means by Student-Newman-Keulz test. A value of *p* < 0.05 was considered significant.

## 3 RESULTS

**Fig. (1):**
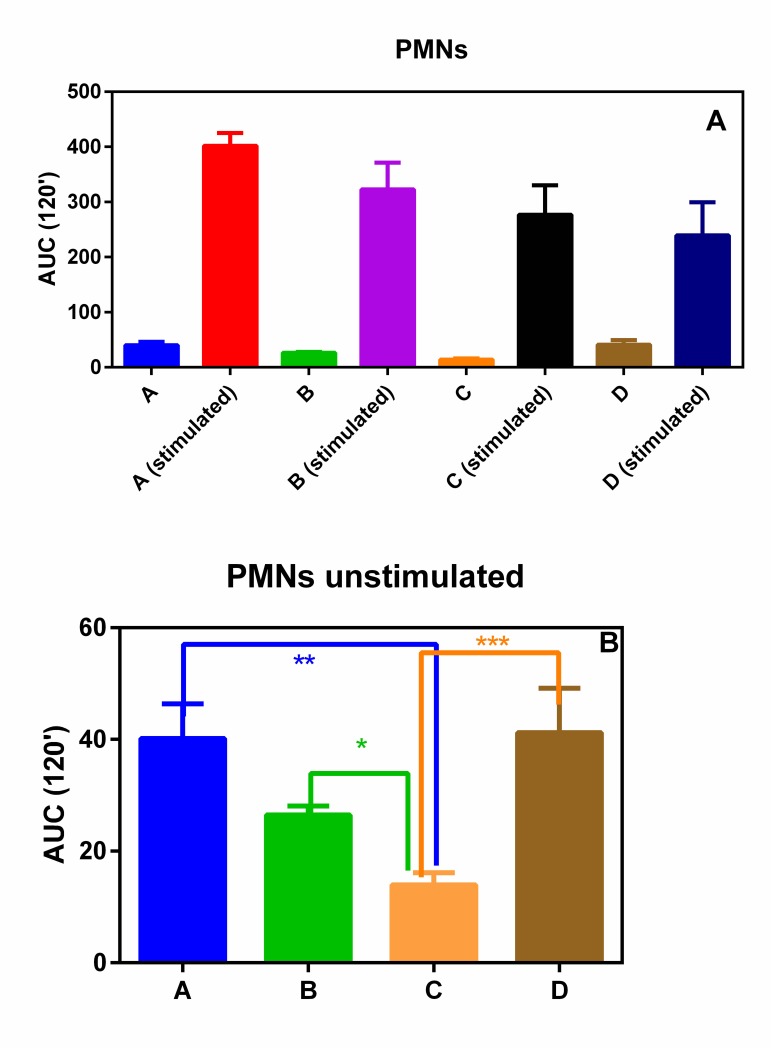
**panel A** CL integral (0-2 h) of PMNs in presence or absence of PMA; **Panel B** CL integral (0-2 h) of unstimulated PMNs. *p< 0.05**p < 0.01; ***p < 0.001.

PMNs and PBMs isolated from all the subjects involving in this pilot study showed a similar oxidative burst (Figs. **1A** and **1B**). In fact, no statistical differences were observed among the four groups.

**Fig. (2):**
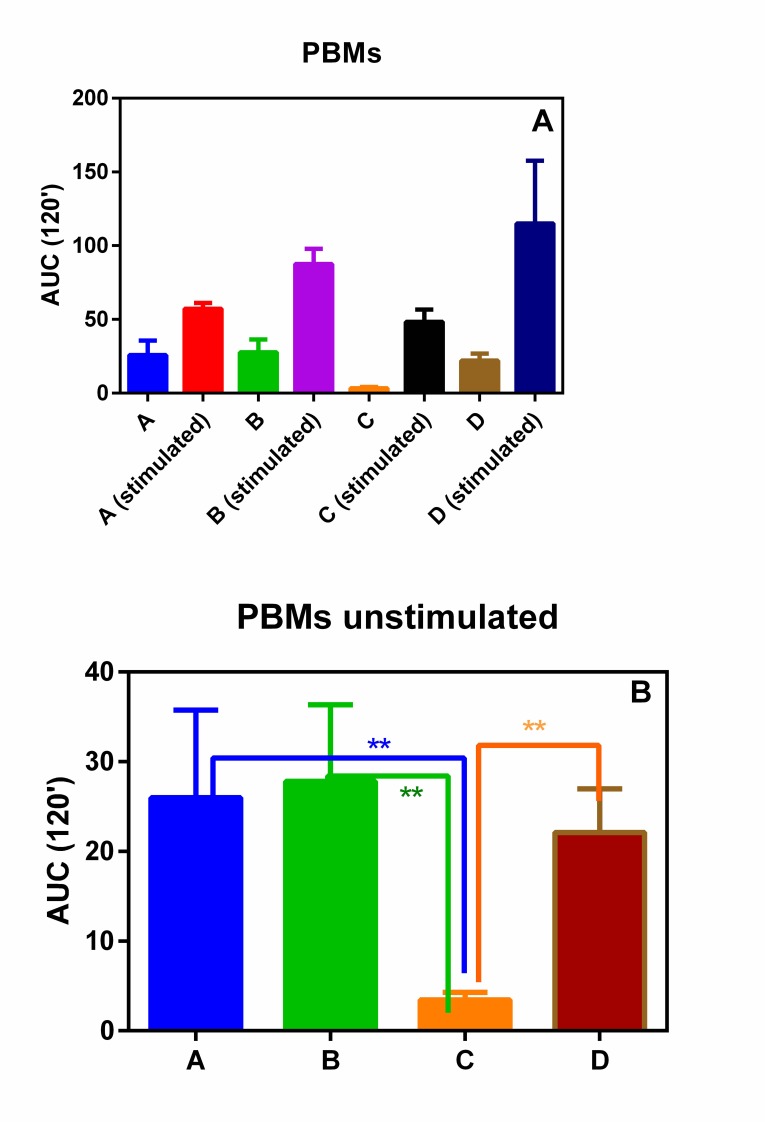
**panel A** CL integral (0-2 h) of PBMs in presence or absence of PMA; **Panel B** CL integral (0-2 h) of unstimulated PBMs.**p < 0.01.

On the contrary, when unstimulated ROS production was considered, group C (normal weight subjects without any form of periodontal disease) showed a statically reduced production of ROS with respect to all the other three groups (Figs. **2A** and **2B**).

## 4 DISCUSSION

It is well-known that both MetS and periodontitis are related to pro-inflammatory cytokines production. Among other functions, these molecules are able to active p47phox and, consequently, the membrane NADPH oxidase, an enzyme present on the plasmatic membranes of PMNs and PBMs with the aim to initiate ROS production.

ROS are strong oxidative molecules involved in inflammation process as well as in chronic diseases, but at - physiological concentrations, they act as second messengers to regulate mitosis, apoptosis, cell differentiation, *etc*., [13]. Therefore the need to control the amount of ROS produced by NADPH oxidase is evident.

Since ROS production is involved both in MetS and periodontitis, the results obtained in this pilot study are intriguing and they deserve to be further evaluated.

In fact, we observed that the basal ROS production in PMNs and PBMs, isolated from groups A, B and D with respect to normal weight subjects without any form of periodontal disease (group C) statistically increased. This phenomenon, although not unexpected, is interesting and could help to explain the relationship between MetS and severity of periodontitis observed by Li *et al.* [10].

Only patients with mild form of periodontal disease were involved.

This methodological choice was dictated by the will of the authors to study the molecules involved in the onset of periodontal disease. Li *et al*. [10] demonstrated that in mice, metabolic syndrome provides an enhancement of the destructive effects of periodontitis, but until now there have been no studies in literature investigating such association with humans by evaluating the ROS involvement or hypothesizing the action of other molecules.

A substantial limitation of this study is that the sample of recruited patients is quite small since, in order to avoid any confounding factor, patients suffering from chronic inflammatory or autoimmune diseases, cystic, benign, and malignant lesions in oral cavity or who underwent surgical interventions were excluded. For the same reason, patients with type 2 diabetes mellitus were not considered; since the majority of patients affected by metabolic syndrome quickly develop insulin resistance and type 2 diabetes mellitus is easy to understand that this exclusion criterion strongly compressed the sample size.

## CONCLUSION

Obviously a study with few patients, like this, cannot provide conclusive results. However, it has provided indications that can be confirmed by recruiting many more patients who should be strictly homogeneous from a medical and dental point of view in order to avoid selection bias that could lead to not accurate conclusions.

## Figures and Tables

**Table 1:** Demographic characteristics of patients included in this study.

	Sex	Age (years)	BMI (kg/m^2^)	PSR Code	Systemic Diseases	Smoking Habits	Group
Patient 1	F	45	44,4	0	Absence	Absence	A
Patient 2	M	54	42,3	1	Absence	Ex-smoker	A
Patient 3	M	43	45,1	3	Absence	Absence	B
Patient 4	F	44	41,4	3	Absence	Absence	B
Patient 5	M	55	24,9	0	Absence	Absence	C
Patient 6	F	52	23,2	1	Absence	Absence	C
Patient 7	M	50	22,9	0	Absence	Absence	C
Patient 8	M	50	25,3	0	Absence	Ex-smoker	C
Patient 9	F	51	21,1	3	Absence	Absence	D
Patient 10	M	49	24,9	3	Absence	Ex-smoker	D
Patient 11	M	58	23,3	3	Absence	Ex-smoker	D

F = female; M = male
